# Stress-related expression of the chloroplast EGY3 pseudoprotease and its possible impact on chloroplasts’ proteome composition

**DOI:** 10.3389/fpls.2022.965143

**Published:** 2022-07-22

**Authors:** Małgorzata Adamiec, Jędrzej Dobrogojski, Łukasz Wojtyla, Robert Luciński

**Affiliations:** ^1^Department of Plant Physiology, Faculty of Biology, Institute of Experimental Biology, Adam Mickiewicz University in Poznań, Poznań, Poland; ^2^Department of Biochemistry and Biotechnology, Faculty of Agronomy, Horticulture and Bioengineering, University of Life Sciences, Poznań, Poland

**Keywords:** *Arabidopsis thaliana*, chloroplast, EGY3, high light stress, high-temperature stress, pseudoprotease

## Abstract

The EGY3 is a pseudoprotease, located in the thylakoid membrane, that shares homology with the family of site-2-proteases (S2P). Although S2P proteases are present in the cells of all living organisms, the EGY3 was found only in plant cells. The sequence of the pseudoprotease is highly conserved in the plant kingdom; however, little is known about its physiological importance. Results obtained with real-time PCR indicated that the expression of the *EGY3* gene is dramatically induced during the first few hours of exposure to high light and high-temperature stress. The observed increase in transcript abundance correlates with protein accumulation level, which indicates that EGY3 participates in response to both high-temperature and high light stresses. The lack of the pseudoprotease leads, in both stresses, to lower concentrations of hydrogen peroxide. However, the decrease of chloroplast copper/zinc superoxide dismutase 2 level was observed only during the high light stress. In both analyzed stressful conditions, proteins related to RubisCO folding, glycine metabolism, and photosystem I were identified as differently accumulating in *egy3* mutant lines and WT plants; however, the functional status of PSII during analyzed stressful conditions remains very similar. Our results lead to a conclusion that EGY3 pseudoprotease participates in response to high light and high-temperature stress; however, its role is associated rather with photosystem I and light-independent reactions of photosynthesis.

## Introduction

Pseudoenzymes are proteins sharing sequence homology with enzyme families but proven or predicted to be inactive due to mutations in amino acid motives crucial for catalytic activity. They are present in all kingdoms of life and are conserved in numerous protein families (Murphy et al., 2017). The pseudoenzymes are known to have divergent functions. Many of them were proven to participate in the allosteric regulation of conventional enzymes (Lingaraju et al., 2014; Rosenberg et al., 2015), others serve as a scaffold for the assembly of enzyme complexes (Fulcher et al., 2018; Walden et al., 2018). Some pseudoenzymes are involved in positioning the active enzyme in the proximity of its substrate (Schäfer et al., 2014; Murphy et al., 2015; Rosenberg et al., 2015) and regulation of protein localization in a cell (Ng et al., 2013; Schroeder et al., 2014). Despite the growing amount of data, the knowledge about the functions and molecular action mechanisms of the pseudoenzymes remains very elusive, and the role of many of them remains unknown or poorly investigated. One of the pseudoenzymes, whose physiological importance remains poorly understood is the ethylene-dependent gravitropism-deficient and yellow-green-like 3 (EGY3) protein. The EGY3 belongs to the pseudoprotease class and shares homology with the family of site-2-proteases (S2P). These proteases are unusually hydrophobic integral membrane zinc-metalloproteases able to perform the proteolytic cleavage within the cell membrane. They are present, among others, in prokaryotic, mammalian, and plant cells. In general, they are involved in the process called regulated intramembrane proteolysis (RIP) by performing, within the cell membrane, the proteolytic cleavage of membrane-anchored transcription factors (Adamiec et al., 2017). S2P proteases were proven to participate in many different physiological processes. In prokaryotic cells, they are involved in pathogenesis, stress response, and sporulation, and in mammalian cells, they were proven to regulate lipid metabolism (Adamiec et al., 2017). In *Arabidopsis thaliana* five S2P proteolytically active proteases and EGY3 pseudoenzyme have been identified. Only one protease, encoded by the *AT4G20310* gene, was found to be located in the Golgi membrane (for review see Adamiec et al., 2017). Four of the proteases, namely EGY1, EGY2, S2P2, and ARASP, were experimentally confirmed to be located in chloroplasts. Also, the EGY3 pseudoenzyme was experimentally confirmed to be located in the chloroplast thylakoid membrane (Adamiec et al., 2019). The role of S2P in plants is poorly investigated. The lack of EGY1 protease leads in *A. thaliana* to pleiotropic effects, such as yellow-green, early senescence phenotype, deficiency in ethylene-induced gravitropism, or oversensitivity to ammonium stress (Chen et al., 2005; Guo et al., 2008; Yu et al., 2016). Also, the *Arabidopsis* serine protease (ARASP) is crucial for *A. thaliana* development since it lacks leads to emerging small, red cotyledons, underdeveloped roots, no apical meristem, and a life expectancy of fewer than 20 days (Bölter et al., 2006). Even less is known about the only pseudoenzyme homologous to this protease family – the EGY3 protein. This pseudoprotease was identified only in plant cells and remains highly conserved in the plant kingdom. In plants, grown in standard laboratory conditions, the lack of the protease does not lead to any visible phenotype changes; however, in *egy3* mutants, the increased value of the non-photochemical quenching and slower recovery rate after photoinhibitory treatment were observed (Adamiec et al., 2019). It has been also suggested that EGY3 may participate in response to salt stress by promoting the copper/zinc superoxide dismutase 2 (CSD2) stability and H_2_O_2_-mediated chloroplastic retrograde signaling (Zhuang et al., 2021). The transcriptional data indicate also that expression of the gene encoding EGY3 is significantly increased under the high-temperature treatment. These data suggest that EGY3 protein may be involved in the response to high light or high-temperature stresses; however, the knowledge on this subject remains limited only to a few observations. We decided to investigate this issue.

## Materials and methods

### EGY3 T-DNA insertion mutants

Two commercially available mutant lines with a T-DNA insertion in the *At1g17870* encoding the EGY3 protein were obtained from NASC (Nottingham Arabidopsis Stock Centre, Nottingham, United Kingdom) and used: SALK_128120 described as *egy3-1* and SALK_042231 described as *egy3-2*. The homozygosity of both lines was previously confirmed with the PCR technique, and the lack of the EGY3 protein was confirmed with the use of an anti-EGY3 antibody (Adamiec et al., 2019).

### Growth and stress conditions

Wild-type (WT) and *A. thaliana* (L.) Heynh (ecotype Columbia) as well as *egy3-1* and *egy3-2* mutant lines were grown on sphagnum peat moss and wood pulp in 42-mm Jiffy peat pellets (AgroWit, Przylep, Poland) under photoperiod 16 h of light/8 h of darkness at an irradiance of 110 μmol m^−2^ s^−1^, relative humidity of 70%, and constant temperature of 22°C for 4 weeks.

The high-temperature stress had been applied by transferring the 4-week plants to 40°C for 1, 3, 6, and 24 h, with the maintained photoperiod.

For the light stress, the plants were exposed to continuous light of intensity 1,000 μmol m^−2^ s^−1^ for 1, 3, 6, and 24 h.

### *EGY3* gene expression analysis

Total RNA from *A. thaliana* leaves (WT) was isolated using the GeneMATRIX Universal RNA Purification Kit (EURX^®^, Poland), according to the manufacturer’s protocol. Isolated RNA was treated with RNase-free DNase (Thermo Fisher Scientific, Waltham, United States), in accordance with the manufacturer’s instruction. Reverse transcription was performed using the RevertAid H Minus First Strand cDNA Synthesis Kit (Thermo Fisher Scientific, Waltham, United States) with random hexamers as primers and 5 μg of total RNA. The quantitative real-time PCR was performed according to Pietrowska-Borek et al. (2020) using the CFX96 Real-Time PCR Detection System (Bio-Rad, Hercules, United States) and iTaq Universal SYBR Green Supermix (Bio-Rad, Hercules, United States). The reaction was carried out in a total volume of 20 μl with 1 μl of cDNA. For relative *EGY3* gene expression quantification in plants exposed to heat or high light stress, the comparative C*_T_* method was used with the aldehyde dehydrogenase 3 (*ALDH3*, *At4g34240*) (for heat stress variant) and the cyclophilin 5 (*CYP5*, *At2g29960*; for high light stress) as the endogenous control. The amount of target normalized to an endogenous control is given by 2^−ΔΔCT^. The primers for the *EGY3, ALDH3*, and *CYP5* genes are as follows:

*EGY3:*

Forward: 5´-GCCCGTCGTTTCTTGTGCCATC-3´.

Reverse: 5´-AAGCAGAAGCGAGGTCAGGTAC-3´.

*ALDH3:*

Forward: 5´-GCAGCGTATCTCTTCACAAACAAC-3´.

Reverse: 5´-ATCCCACTCTCCCCAACCCCAC-3´.

*CYP5:*

Forward: 5′-GAGAAAGGTGTAGGGAAGAGTGG-3´.

Reverse: 5´-CAAACTTCTGACCATAGATTGATTC-3´.

All primers were tested for non-specific amplification and primer-dimer formation by melting curve analysis. For each sample, three biological repetitions were performed, each in three technical repetitions.

### Total leaf protein isolation

For single isolation, 100 mg of *A. thaliana* leaf tissue was used. The isolation of total leaf protein was performed with the use of Protein Extraction Buffer (PEB, Vannas, Agrisera), according to the manufacturer’s instructions. The concentration of the extracted protein was measured with Lowry et al. (1951) method with the Lowry DC kit (Bio-Rad, Hercules, CA, United States).

### SDS-page and immunoblotting

The SDS-PAGE was performed, according to Laemmli (1970), with the use of 12% (w/v) polyacrylamide gels containing 6 M urea (Sigma-Aldrich, St. Louis, United States). After the separation, proteins were transferred to PVDF membranes (Bio-Rad, United States) and a standard western blot procedure was applied (Adamiec et al., 2018). The PVDF membrane was blocked using 4% BSA (BioShop, Burlington, Canada) and incubated with specific, primary antibodies for 90 min. Next incubation with a secondary antibody (Agrisera, Vannas, Sweden) was performed and the relevant bands were digitally registered using the ChemiDoc™MP Imaging System (Bio-Rad, Hercules, CA, United States) after 5 min of incubation with the Clarity Western ECL Substrate (Bio-Rad, Hercules, CA, United States). Quantification of the immunostained bands was performed using GelixOne software (Biostep GmbH, Jahnsdorf, Germany). Only blots with a linear relationship between the strength of the signal and the amount of protein were analyzed. The linearity of the signal was investigated in our previous work (Adamiec et al., 2018).

### Antibodies

The highly purified N-terminal region (AA 51–250) of EGY3 from *A. thaliana* was used to produce the specific, polyclonal, rabbit anti-EGY3 antibody. The antibody was custom produced by Agrisera and their specificity has been described by us earlier (Adamiec et al., 2019). Anti-PsaB, anti-GLDP, anti-CSD2, and secondary antibodies were purchased from Agrisera (Vännäs, Sweden).

### Chlorophyll fluorescence measurements

Chlorophyll fluorescence measurements were conducted using FMS1 (Photon System Instruments, Brno, Czech Republic) run by Modfluor software. Each measurement was preceded by adaptation in the dark for 30 min. The measurements were performed according to the protocol described by Genty et al. (1989). The minimum fluorescence yield (F_0_) was established at the beginning of the measurement. The maximum quantum yield of PSII (F_v_/F_m_) and quantum efficiency of open centers in the light (F_v_′/F_m_′) were calculated according to Genty et al. (1989). The applied actinic light intensity was equal to the irradiance before dark-adaptation: 110 μmol m^−2^ s^−1^ for control conditions and plants exposed to high-temperature stress and 1,000 μmol m^−2^ s^−1^ for plants exposed to high light. The photochemical quenching (qP), a photochemical yield of photosystem II (ΦPSII) as well as the non-photochemical quenching parameter (NPQ) were calculated according to Maxwell and Johnson (2000). Ten plants from each variant (*WT, egy3-1, and egy3-2*) were measured in each replicate.

### 2D Electrophoresis and LC–MS/MS analysis

#### Chloroplast isolation and fractioning

Chloroplasts were isolated according to our previous work (Adamiec et al., 2018) using the Sigma Chloroplast Isolation Kit (Sigma- Aldrich, St. Louis, United States). For single isolation, 20 g of *A. thaliana* leaf tissue was used. The tissue was homogenized in an ice-cold homogenization buffer with the addition of 1% (v/v) Protease Inhibitor Cocktail (PIC; Sigma-Aldrich, St. Louis, United States). The homogenate was filtered through a Mesh 100 filter and centrifuged at 200 *g* for 1 min at 4°C to remove the unbroken cells. Next, the centrifugation at 1,500 *g* for 10 min at 4°C was performed to sediment the chloroplasts and then resuspended in the homogenization buffer with 1% (v/v) PIC. Subsequently, the intact chloroplasts were obtained, as a pellet, by centrifugation of the chloroplast suspension through 40% (w/v) Percoll for 6 min at 1,700 *g*. To separate the stroma and the thylakoid membranes, the intact chloroplasts were resuspended in the lysis buffer with the addition of 1% (v/v) PIC and centrifuged for 10 min at 12,250 *g*. Both the supernatant containing stroma and a green pellet containing the thylakoid membranes were frozen in liquid nitrogen, stored at −80°C, and then used for protein extraction.

#### Protein extraction from the thylakoids and the stroma

Slightly different procedures were used to extract protein from thylakoids and stroma. For extract protein from thylakoids, the thylakoids membranes were homogenized in 4°C with the EB buffer (Tris–HCl pH 7.5, 25% (w/v) sucrose, 5% glycerol (v/v), 10 mM EDTA, 10 mM EGTA, 5 mM KCl, and 1 mM DTT) with the addition of 0.5% (w/v) PVPP and 1% (v/v) PIC to avoid proteolysis. Subsequently, centrifugation was performed at 600 *g* for 3 min. The supernatant was diluted 2-times with water to reach a 12% concentration of sucrose in the EB buffer and centrifuged for 60 min at 100,000 *g*. The pellet was resuspended in the Tris–HCl buffer (pH 7.5) containing 5 mM EDTA and EGTA and 1% (v/v) PIC. The Bradford method (Bradford, 1976) was used to measure the protein concentration, and then, proteins were solubilized in the presence of 2% (w/v) Brij^®^ 58 (Sigma-Aldrich, St. Louis, United States) for 1 h at 4°C and precipitated with acetone with 10% (w/v) TCA and 0.07% (v/v) β-mercaptoethanol overnight at −20°C. After the precipitation, the proteins were pelleted by centrifugation for 15 min at 20,000 *g*, washed three times with pure acetone, and resuspended in a buffer containing 7 M urea, 3 M thiourea, 2% (w/v) amidosulfobetaine-14 (ASB-14; Sigma-Aldrich, St. Louis, United States), and 65 mM DTT for 2 h at room temperature with constant, gentle shaking and then applied for isoelectrofocusing (Adamiec et al., 2018).

The extraction of stroma proteins consisted of their precipitation with 3 volumes of acetone with the addition of 10% (w/v) TCA and 0.07% (v/v) β-mercaptoethanol overnight at −20°C. The sequential steps of washing the proteins and suspending them in the buffer were identical to those for the thylakoid proteins, except that 2% ASB in the buffer was replaced with 4% CHAPS (Sigma-Aldrich, St. Louis, United States).

### Isoelectrofocusing and spot detection

Isoelectrofocusing was carried out using the gel strips forming an immobilized pH gradient from 3 to 10 (Bio-Rad, Hercules, CA, United States). Strips were rehydrated overnight at room temperature and the isoelectrofocusing was performed at 18°C in the Protean i12 IEF Cell (Bio-Rad, Singapore) for 90 min (for thylakoid membrane proteins) or 60 min (for stroma proteins) at 300 V, 90 min at 3,500 V, and 20,000 Vh at 5,500 V. After the IEF strips were equilibrated according to Kubala et al. (2015) and the proteins were separated according to their molecular mass using denatured electrophoresis in 12% (w/v) acrylamide gels with the addition of 6 M urea. After electrophoresis, the gels were stained with Coomassie Brilliant Blue (CBB) G-250 and photographed with a ChemiDoc™MP Imaging System (Bio-Rad, Hercules, CA, United States). Finally, four images, representing two independent biological replicates, were obtained and used for the image analysis.

The spot detection and the image analysis were performed according to our previous work (Adamiec et al., 2018), using the PDQest Advanced 2-D Gel Analysis Software (Bio-Rad, Hercules, United States). Only the differentially accumulated proteins (at P<0.05) between WT and *egy3* mutant lines with the ratio of at least 2.0 in the absolute value of protein abundance were taken into consideration and further analyzed. The selected spots were excised manually and analyzed by liquid chromatography coupled to the mass spectrometer in the Laboratory of Mass Spectrometry, Institute of Biochemistry and Biophysics, Polish Academy of Science (Warsaw, Poland), according to the previous description by Kubala et al. (2015). The Mascot Distiller software was used to process the raw data, and then, the obtained protein masses and fragmentation spectra were matched TAIR filter with the use of the Mascot Daemon engine search. The search parameters were set as described previously (Adamiec et al., 2018). Only the peptides with a Mascot score exceeding the threshold value corresponding to < 0.05 false-positive rate have been considered as positively identified.

### Detection of hydrogen peroxide

3,3′-diaminobenzidine (DAB) staining was performed according to Daudi and O’Brien (2012). Rosette leaves of the plants treated with abiotic stress and control plants were incubated in the DAB staining solution (10 mM Na_2_HPO_4_, 0.05% [v/v] Tween, 1 mg/ml DAB, pH: 7.4) overnight. Later, the chlorophyll was cleared by boiling the leaf in a bleaching solution (3 ethanol: 1 acetic acid: 1 glycerol) for 20 min. The samples were photographed using a ChemiDoc™MP Imaging System (Bio-Rad, Hercules, CA, United States).

Quantification of H_2_O_2_ levels with DAB was performed according to Boyidi et al. (2021) with modifications. The DAB-stained leaves have been weighed, homogenized, extracted in 2 ml on 1 mg of fresh tissue of perchloric acid, and centrifuged at 10,000 *g* for 10 min. Next, the absorbance was measured at 450 nm. The H_2_O_2_ levels were represented as μmol/g FW.

Spectrophotometric determination of hydrogen peroxide was performed based on the titanium (Ti^4+^) method according to Becana et al. (1986). *Arabidopsis thaliana* leaves (0.25 g) were homogenized in 3 ml of 100 mM phosphate buffer pH 7.8 with the addition of active charcoal at a proportion of 1:5. The homogenate was centrifuged at 15,000 *g* for 30 min at 4°C. For spectrophotometric measurement, the reactive mixture containing 100 mM phosphate buffer pH 7.8, plant extract, and the titanium reagent consisting of 0.3 mM 4-(2-pyridylazo) resorcinol and 0.3 mM titanium potassium tartrate at the ratio 1:1 was prepared in spectrophotometric cuvettes. The absorbance was measured at 508 nm against the calibration curve prepared for the standards containing H_2_O_2_ from 0 to 100 nmol. The accumulation of H_2_O_2_ was expressed as an amount of H_2_O_2_ in 1 g of FW.

## Results

### The changes of *EGY3* expression level in response to high light and high-temperature stresses in wild-type *Arabidopsis thaliana* plants

**Figure 1:**
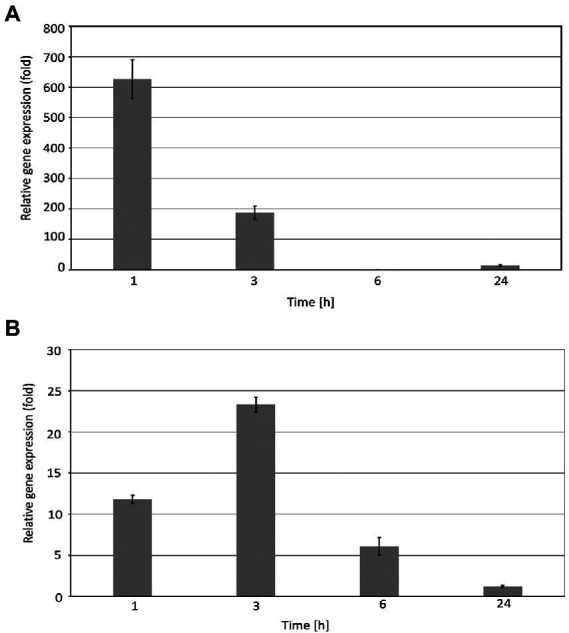
Relative *EGY3* gene expression in *Arabidopsis thaliana* leaves exposed to heat stress **(A)** or high light **(B)**. *Arabidopsis thaliana* plants were exposed to 1,000 μmol m^−2^ s^−1^ or for 40°C for 1 h, 3 h, 6 h, and 24 h, and then, the total RNA was isolated from leaves and transcribed into cDNA used as a template for the quantitative real-time PCR, according to the description in the Material and Methods. *CYP5* and *ALDH3* were used as an endogenous control for the analysis of plants exposed to high light or heat stress, respectively. ± means standard deviation of six replication, an asterisk indicates a statistically significant change compared to the 0 h time base value.

The real-time PCR experiments revealed that the expression level of the *EGY3* gene is dramatically induced during exposure to the high-temperature stress. The strongest induction of transcription was observed in the initial stages of stress. After 1 h of the exposition of WT plants to 40°C, the relative expression of the *EGY3* gene was induced about 630-fold. The longer exposure to stressful conditions (for 3 h) resulted in a 190-fold change increase in the level of transcription, and after the 6 h of high-temperature stress, the *EGY3* expression was similar to the one in control conditions. After 24 h of plant exposure to high temperature, the increase in *EGY3* expression level was relatively smaller concerning changes observed during the first hours of exposition to stressful conditions and represented 13-fold change concerning the initial level (Figure 1A). Also, during the exposition to high light stress, the increase in the relative expression of the *EGY3* gene was observed, and similarly to high-temperature stress, the most significant increase was detected at the initial stages of exposition to stress. The 1 h of high light stress resulted in 11-fold increase of *EGY3* transcription and 3 h of elevated irradiance caused 23-fold up-regulation. After 6 h of exposition to high light, a 6-fold increase in *EGY3* relative expression level was observed, while after 24 h of high light stress, the abundance of transcript remained at the level similar to control conditions (Figure 1B).

### The changes of EGY3 protein abundance in response to high light and high-temperature stresses in wild-type *Arabidopsis thaliana* plants

**Figure 2:**
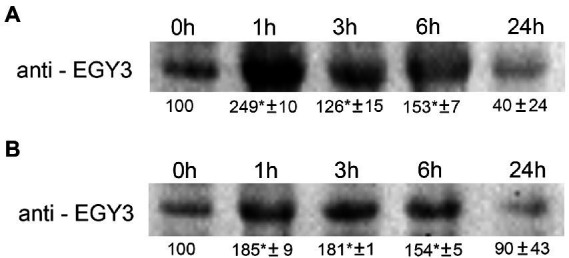
Immunoblot quantification of EGY3 protein in wild-type plants (WT) under high-temperature stress **(A)** and during an exposition to high light conditions **(B)**. The plants were exposed for 1 h, 3 h, 6 h, and 24 h for 40°C to cause high-temperature stress and for 1,000 μmol m^−2^ s^−1^ to cause high light stress. “±” means standard deviation, the asterisk means the significant difference in comparison to 0 h time (no stress).

The changes observed at the gene expression level correlated with changes in protein abundance. During the high-temperature stress, the increase in protein abundance was highest after 1 h and amounted to 250% of the control level. The protein overaccumulated also after 3 and 6 h exposition to a high temperature to 126 and 150% of initial values. The 24 h long exposure to high temperature resulted in a decrease in protein accumulation level to 40% concerning control conditions (Figure 2A).

The 1 h exposition to high light resulted in a 185% increase in protein abundance. A similar level (180%) was observed after 3 h of exposition and the application of stressful conditions for 6 h increased to 150% of the initial value. The 24 h of exposition to increased irradiance did not result in significant changes in EGY3 abundance (Figure 2B).

### Comparative analysis of the chloroplast proteome

**Figure 3:**
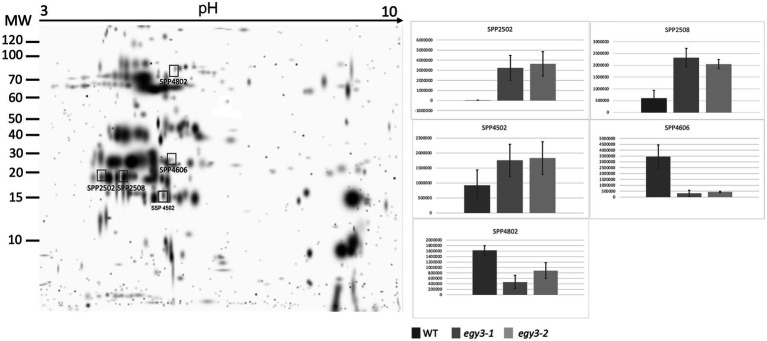
Two-dimensional electrophoresis gels of thylakoid membrane proteins isolated from wild-type (WT) and *egy3-1* and *egy3-2* lines, exposed to 3 h of high light conditions. Proteins were separated using 2-D gel electrophoresis with IEF (pH 3–10) and detected with Coomassie Brilliant Blue staining. The image is digitally generated using the PDQuest Advanced 2-D Gel Analysis Software, (Bio-Rad, Hercules, United States) master gel, based on four electrophoretic separations made for two independent biological repetitions. The protein accumulation in the bar graphs was expressed in arbitrary units.

**Figure 4:**
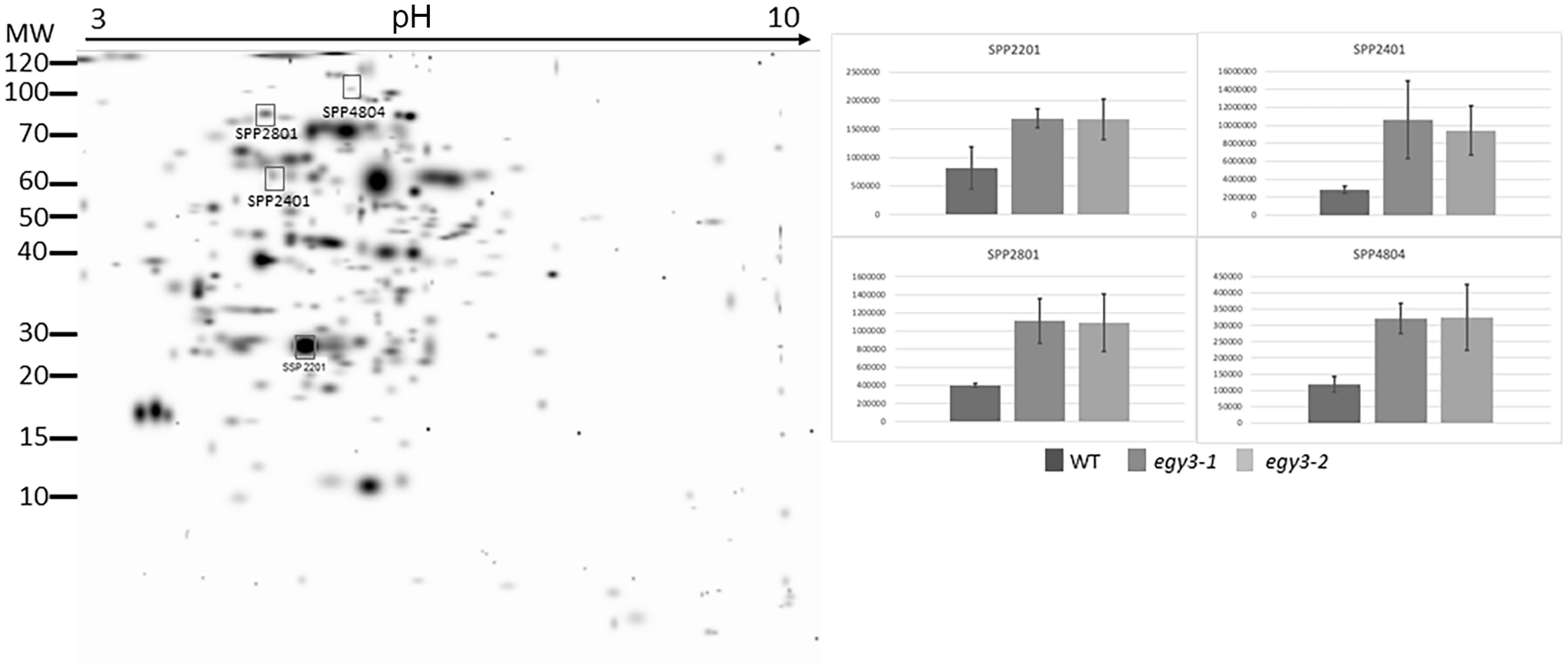
Two-dimensional electrophoresis gels of stroma proteins isolated from wild-type (WT) and *egy3-1* and *egy3-2* lines, exposed to 3 h of high light conditions. Proteins were separated using 2-D gel electrophoresis with IEF (pH 3–10) and detected with Coomassie Brilliant Blue staining. The image is digitally generated using the PDQuest Advanced 2-D Gel Analysis Software, (Bio-Rad, Hercules, United States) master gel, based on four electrophoretic separations made for two independent biological repetitions. The protein accumulation in the bar graphs was expressed in arbitrary units.

**Figure 5:**
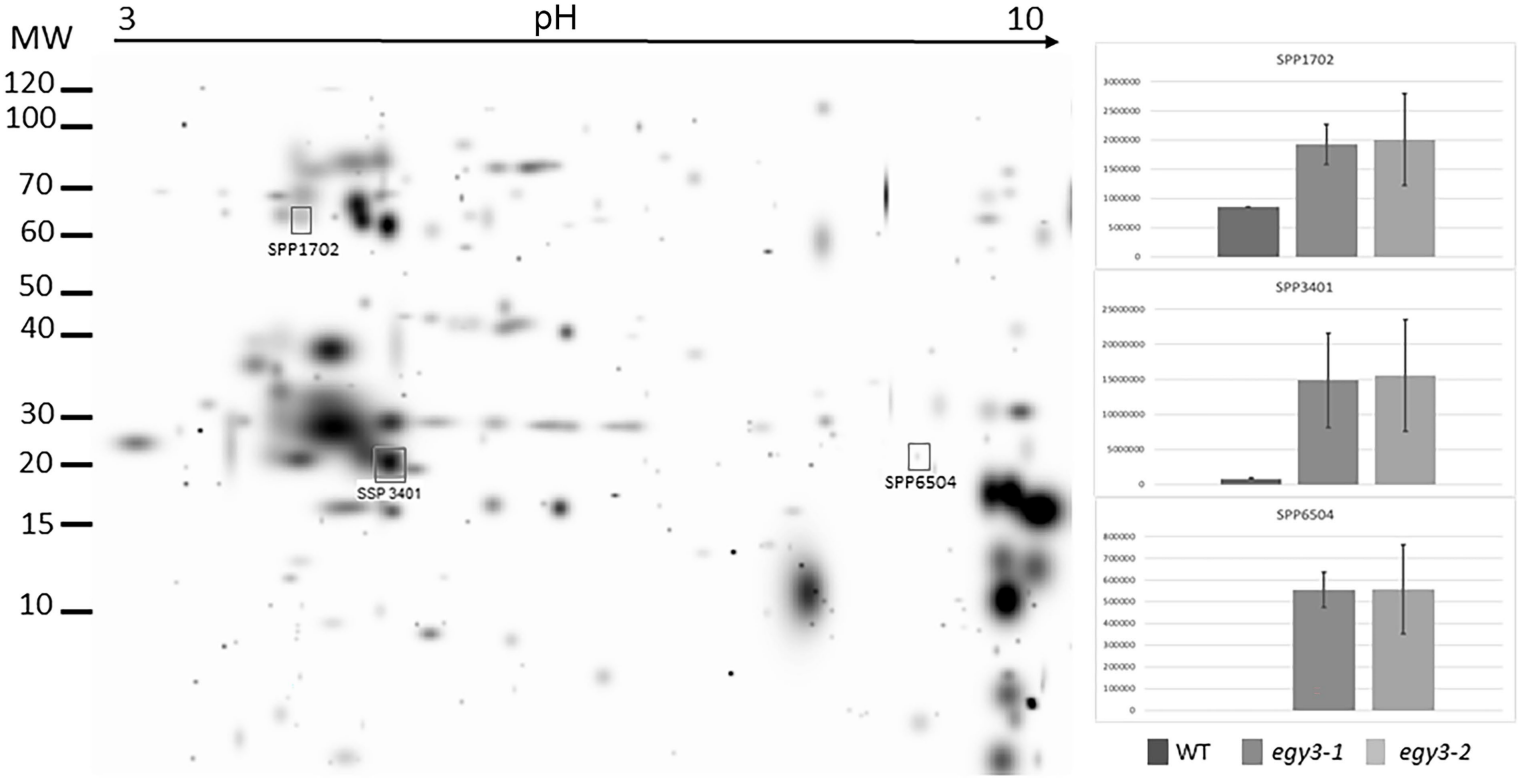
Two-dimensional electrophoresis gels of thylakoid membrane proteins isolated from wild-type (WT) and *egy3-1* and *egy3-2* lines, exposed to 3 h of 40°C. Proteins were separated using 2-D gel electrophoresis with IEF (pH 3–10) and detected with Coomassie Brilliant Blue staining. The image is digitally generated using the PDQuest Advanced 2-D Gel Analysis Software, (Bio-Rad, Hercules, United States) master gel, based on four electrophoretic separations made for two independent biological repetitions. The protein accumulation in the bar graphs was expressed in arbitrary units.

**Figure 6:**
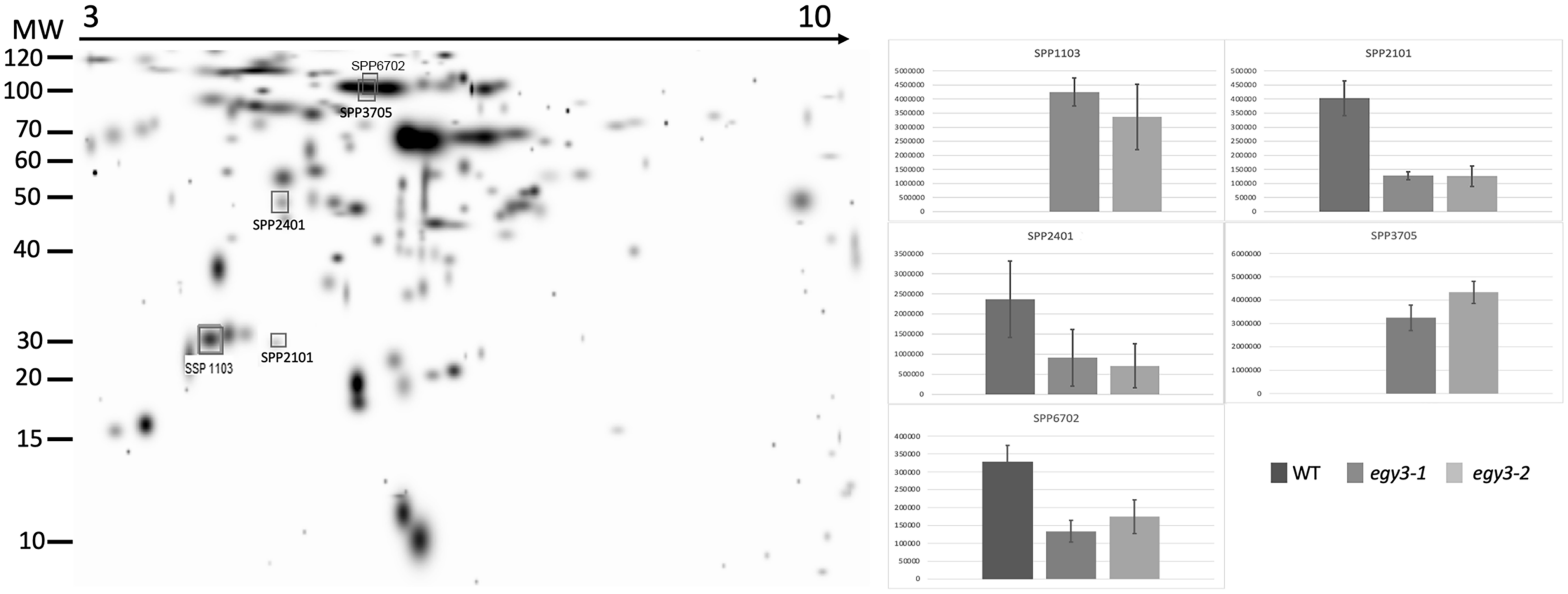
Two-dimensional electrophoresis gels of stroma proteins isolated from wild-type (WT) and *egy3-1* and *egy3-2* lines, exposed to 3 h of 400°C. Proteins were separated using 2-D gel electrophoresis with IEF (pH 3–10) and detected with Coomassie Brilliant Blue staining. The image is digitally generated using the PDQuest Advanced 2-D Gel Analysis Software, (Bio-Rad, Hercules, United States) master gel, based on four electrophoretic separations made for two independent biological repetitions. The protein accumulation in the bar graphs was expressed in arbitrary units.

Based on previous analysis concerning changes in the quantity of *EGY3* transcript and protein, we decided to perform an analysis of changes in the proteome of *egy3* mutants in plants exposed to the elevated irradiance for 3 h. The same exposure time was selected for further research on plants exposed to high temperature. From the WT plants and both *egy3* mutant lines exposed for 3 h to a given stress, the thylakoid membrane fraction and stroma fraction were isolated. Both fractions were subjected to two-dimensional electrophoresis and protein spots whose accumulation level differed at least 2-fold from that observed in wild-type in both *egy3* mutant lines in two separate biological replicates were identified (Figures 3–6). For further analysis by LC–MS/MS, from the high light stress experimental variant, we choose five protein spots from thylakoid fraction and four from the stroma fraction. From the high-temperature experimental variant, three spots from the thylakoid fraction and five from the stroma fraction were chosen and LC–MS/MS analysis was performed. Based on pH, molecular mass parameters, and score parameters, the most probable proteins whose abundance may be EGY3 dependent were selected (Tables 1 and 2).

**Table 1:** The proteins identified as differently accumulating in *egy3* mutants in response for 3 h exposition to high light.

Locus	Protein name	Spot number	Direction of change	Fraction	MW	pI	Protein score		Number of peptide matches		Protein coverage (%)	
					Theor/exper (kDa)	Theor/exper	I	II	I	II	I	II
AT1G15820	Lhcb6	SPP4606	D	T	28/27.5	7.0/6.7	199	138	2	1	10.9	7
ATCG00340	PsaB	SPP4802	D	T	80/82.5	7.0/6.8	205	114	4	1	6.1	1.6
AT4G32260	PDE334	SPP2508	O	T	24/23.9	6/5.8	401	241	5	5	23.3	19.2
AT4G37925	NDH-M	SPP2502	O	T	24/24.9	5/4.8	956	235	13	3	39.6	18.4
ATCG00470	ATPE	SPP4502	O	T	17/14.5	6.5/5.8	40	32	1	1	11.4	8.1
At1G78370	ATGSTU20	SPP2201	O	S	25/25.1	5.5/5.4	812	767	17	14	33.6	33.6
AT4G33010	AtGLDP1	SPP4804	O	S	115/113.8	6.5/6.5	1,216	840	22	18	12.7	12.6
AT2G28000	CPN60A	SPP2401	O	S	60/62.2	5/5.1	628	710	9	11	16.9	18.9
AT5G49910	CPHSC70-2	SPP2801	O	S	80/77	5/5.2	201	675	5	11	7.8	11.8

Selected spots were cut from two gels representing different *egy3* mutant lines and subjected to a separate LC–MS/MS analysis. Only proteins identified in both replicates were taken for further consideration. Based on pH, molecular mass parameters, and score parameters from proteins identified by the LC–MS/MS method we selected the most probable proteins whose abundance may be EGY3 dependent. D – decrease in *egy3*; O – overaccumulation in *egy3*; T – thylakoid fraction (grey highlighted), S – stroma fraction (non-highlighted). The exact location of individual protein spots on polyacrylamide gels after 2-D electrophoresis is shown in Figure 3 (thylakoid fraction) and Figure 4 (stroma fraction).

**Table 2:** The proteins identified as differently accumulating in *egy3* mutants in response for 3 h exposition to high temperature.

Locus	Protein name	Spot number	Direction of change	Fraction	MW	pI	Protein score		Number of peptide matches		Protein coverage (%)	
					Theor/exper (kDa)	Theor/exper	I	II	I	II	I	II
At1G03130	PSAD-2	SPP6504	O	T	20/22.3	9.5/9.8	128	56	2	2	10.8	6.9
AT2G30390	ATFC-II	SPP1702	O	T	58/56.8	5.2/5.1	75	72	1	1	2.3	2.3
ATCG00130	ATPF	SPP3401	O	T	19/21	6.0/6,1	151	108	4	3	23.4	13.6
AT1G16880	ACR11	SPP1103	P	S	30/31.4	4.6/4.9	680	684	10	11	16.9	19.7
AT5G28500	RubisCO accumulation factor-like protein	SPP2401	D	S	45/48.3	4.8/5.0	464	348	8	6	14.5	9.7
AT2G26080	GLDP2	SPP3705	P	S	100/114.7	6.2/6.1	366	336	6	5	6.1	4.6
AT4G33010	GLDP1	SPP6702	D	S	100/113.8	6.2/6.5	395	246	8	5	6.8	4.3
AT3G55440	triosephosphate isomerase	SPP2101	D	S	30/27.4	5,2/5.4	318	110	5	2	22	8.8

Selected spots were cut from two gels representing different *egy3* mutant lines and subjected to a separate LC–MS/MS analysis. Only proteins identified in both replicates were taken for further consideration. Based on pH, molecular mass parameters, and score parameters from proteins identified by the LC–MS/MS method, we selected the most probable proteins which abundance may be EGY3 dependent. D – decrease in *egy3*; O – overaccumulation in *egy3*; P – present in *egy3* but not in WT; T – thylakoid fraction (grey highlighted), S – stroma fraction (non-highlighted). The exact location of individual protein spots on polyacrylamide gels after 2-D electrophoresis is shown in Figure 5 (thylakoid fraction) and Figure 6 (stroma fraction).

Among the protein spots differently accumulating in *egy3* mutants’ thylakoid membranes in response to exposition to high light, two were characterized by a decrease in abundance. Within these spots, the Lhcb6 and PsaB proteins were identified. In the remaining three spots, overaccumulation was observed concerning WT plants. Within these spots, subunit NDH-M of NAD(P)H: plastoquinone dehydrogenase complex, PDE334 protein which is part of proton-transporting ATP synthase complex F(o), and ATP synthase epsilon chain (ATPE) were identified (Table 1). The localization of all these proteins in thylakoid membranes was experimentally confirmed (Friso et al., 2004; Peltier et al., 2004; Rumeau et al., 2005). In the stroma, exposition to high light stress leads to overaccumulation protein in *egy3* mutant lines of all four chosen protein spots. Within these spots, ATGSTU20 protein, which is a glutathione transferase, glycine decarboxylase P-protein 1 (AtGLDP1), CPN60A which encodes chaperonin-60 alpha, and heat shock protein CPHSC70 were identified (Table 1). Also, in this case, stromal localization of these proteins was experimentally confirmed (Peltier et al., 2004; Rutschow et al., 2008; Ferro et al., 2010). The only exception is the ATGSTU20 protein, which localization in chloroplasts is described without indication of the compartment (Zybailov et al., 2008). Predictive algorithms indicate, however, no high hydrophobicity regions in the amino acid sequence of this protein, thus its stroma localization seems highly probable (Schwacke et al., 2003).

In the thylakoid fraction from plants exposed to high temperature, three protein spots, which significantly increased abundance in *egy3* mutant, were chosen for LC–MS/MS. Within these spots, PSAD-2 predicted to be photosystem I reaction center subunit II, ferrochelatase 2 (ATFC-II), and ATPase F subunit (ATPF) proteins were identified (Table 2), and localization of these proteins in the thylakoid membrane was experimentally confirmed (Lister et al., 2001; Peltier et al., 2004). Among the protein spots from stroma fraction, two spots were absent in WT plants but present in both *egy3* mutant lines, and three spots with a lower abundance in *egy3* mutants were chosen. In the spots absent in WT plants, ACT domain-containing protein (ACR11) and glycine decarboxylase P-protein 2 (GLDP2) were identified, while in spots with decreased accumulation, RubisCO accumulation factor-like protein, encoded by *At5g28500* gene, triosephosphate isomerase (TPI), and glycine decarboxylase P-protein 1 (GLDP1) were present (Table 2). The localization of most of these proteins in chloroplast stroma was experimentally confirmed (Peltier et al., 2004; Rutschow et al., 2008; Ferro et al., 2010), except for GLDP2, which was described as located in chloroplast without indication of a compartment (Zybailov et al., 2008); however, the amino acid sequence of the protein lacks highly hydrophobic regions (Schwacke et al., 2003), thus its localization is stroma seems probable.

**Figure 7:**
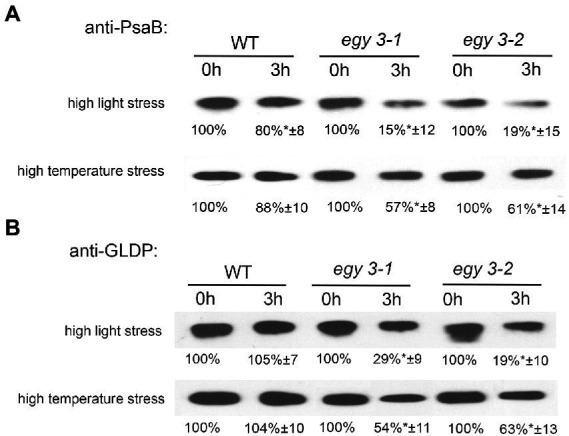
Immunoblot quantification of PsaB **(A)** and GLDP **(B)** proteins in wild-type (WT) and *egy3-1* and *egy3-2* mutant plants under high light and high-temperature conditions. Plants were exposed to 1,000 μmol m^−2^ s^−1^ (high light) or 40°C (high temperature) for 0 and 3 h. Total protein (6 μg) was immunologically analyzed using an anti-PsaB and anti-GLDP antibodies. GelixOne software was used to quantify the protein content. “±” indicates the SD determined in the analysis of samples obtained from three biological replicates, each of which was obtained by isolation of total protein from a minimum of 20 plants.

The changes in abundance of PsaB and GLDP proteins were confirmed with the immunoblot technique (Figures 7A,B). The accumulation level of PsaB protein after 3 h of exposition to high light in WT plants was 80% of the initial state, while in both *egy3* mutant lines, its abundance was reduced to 15% in the *egy3-1* mutant line and 19% in *egy3-2* mutant line. Significantly greater, in WT plants, loss of PsaB was also observed in both *egy3* mutant lines after 3 h exposition to high temperature. In WT plants, the protein level remained at a level of 88%, while in *egy3* mutant lines, its abundance was decreased to 57% in the *egy3-1* mutant line and 61% in the *egy3-2* mutant line (Figure 7A).

The antibody used for the investigation of changes in abundance of the glycine decarboxylase P-protein was specific for both GLDP1 and GLDP2, so the entire pool of GLDP proteins was investigated. The GLDP1 protein was previously detected in both stroma and thylakoid fraction (Ferro et al., 2010), and GLDP2 is described as located in chloroplast without a more specific indication of the compartment (Zybailov et al., 2008). That is why we used for anti-GLDP hybridization experiments the whole leaf protein fraction. In response to high light stress, in both *egy3* mutant lines, the abundance of GLDP proteins was reduced to 20–30%, while in WT plants, its accumulation level remained similar to control conditions. A similar effect was observed after exposition to high-temperature stress; however, the decrease in GLDP abundance was less dramatic – approximately 50–60% of the initial value (Figure 7B). In the WT plants, exposition to high temperature does not cause changes in GLDP accumulation level.

### The changes in abundance of copper/zinc superoxide dismutase 2

**Figure 8:**
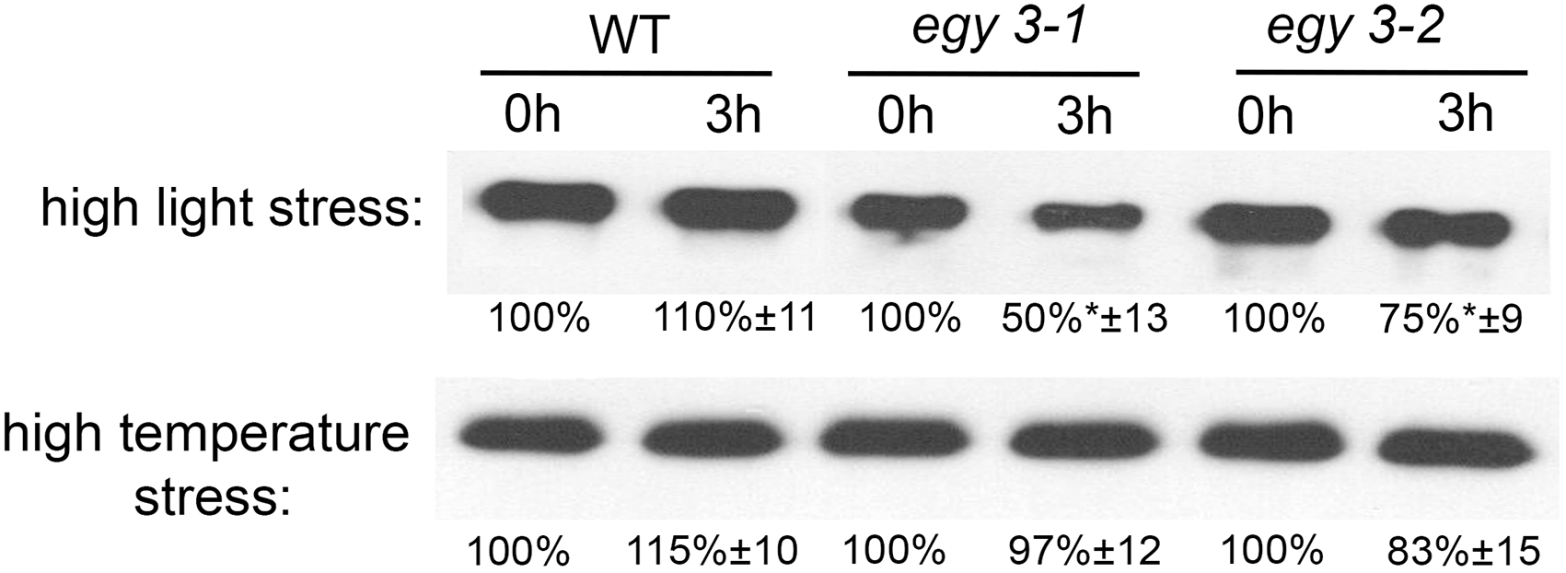
Immunoblot quantification of CSD2 protein in wild-type (WT) and *egy3-1* and *egy3-2* mutant plants under high light and high-temperature conditions. Plants were exposed to 1,000 μmol m^−2^ s^−1^ (high light) or 40°C (high temperature) for 0 and 3 h. Total protein (5 μg) was immunologically analyzed using an anti-CSD2 antibody. GelixOne software was used to quantify the CSD2 content. “±” indicates the SD determined in the analysis of samples obtained from three biological replicates, each of which was obtained by isolation of total protein from a minimum of 20 plants.

Since it was proven that in the salt stress, the EGY3 protein participates in the stabilization of copper/zinc superoxide dismutase 2 (CSD2), which is responsible for the conversion of superoxide anion into hydrogen peroxide and thus is involved in H_2_O_2_–mediated signaling. We decided to investigate the accumulation changes of CSD2 in *egy3* mutants in response to analyzed stresses. Our results indicate that after 3 h of exposition to high light stress, the abundance of CSD2 in WT plants remains similar, while in both *egy3* mutant lines, its accumulation level is significantly decreased (Figure 8). During the high-temperature stress, however, we did not observe significant differences in the accumulation of the CSD2, between WT plants and *egy3* mutant lines (Figure 8).

### The accumulation level of the hydrogen peroxide in *egy3* mutants

**Figure 9:**
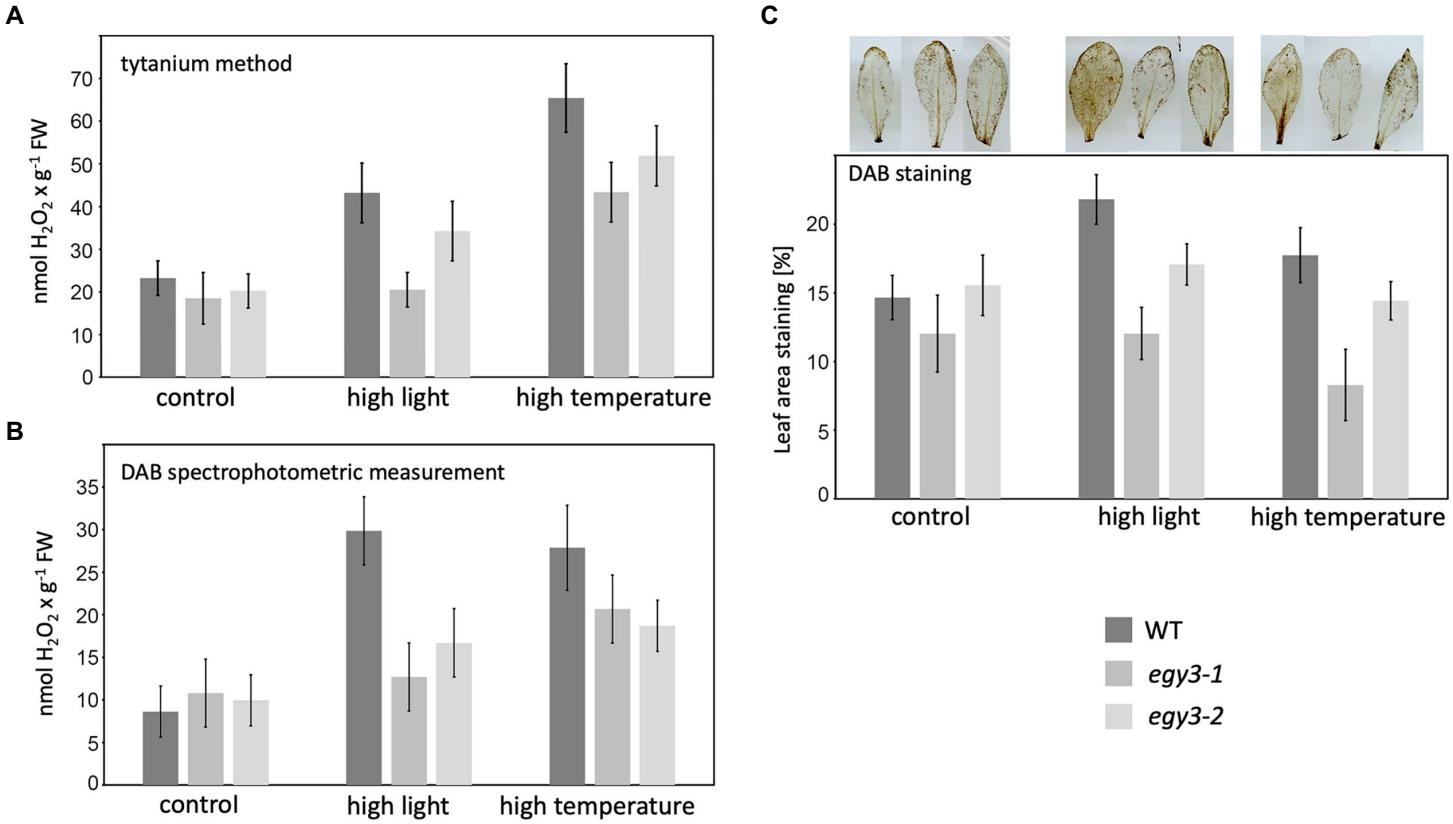
Accumulation of hydrogen peroxide in WT plants and *egy3* mutant lines after 1 h exposition to high light and high-temperature stress. **(A)** Titanium (Ti^4+^) method staining **(B)** DAB staining spectrophotometric measurement **(C)** percentage of leave area staining with DAB. The values presented for Titanium (Ti^4+^) method staining and DAB staining spectrophotometric measurement are average from two biological replicates (10 plants each). The values presented for the percentage of leave area staining with DAB are average from three biological replicates (10 leaves each).

The observed changes in the level of the CSD2 accumulation have become a premise for the determination of the changes in the concentration of hydrogen peroxide in *egy3* mutants in response to analyzed stresses. Three different methods of measurement were applied: a method based on the titanium (Ti^4+^), DAB staining, and spectrophotometric DAB quantification. The results obtained with these three methods were consistent. In both *egy3* mutant lines, in the result of the 1 h exposition to the high light, the increase in H_2_O_2_ concentration was significantly smaller than observed in WT plants (Figures 9A,C). Similarly, exposure to 1 h high-temperature stress leads to a lower, than in WT plants, increase in abundance of hydrogen peroxide (Figures 9B,C).

### The functional status of PSII in *egy3* mutants during high light and high-temperature stress

**Figure 10:**
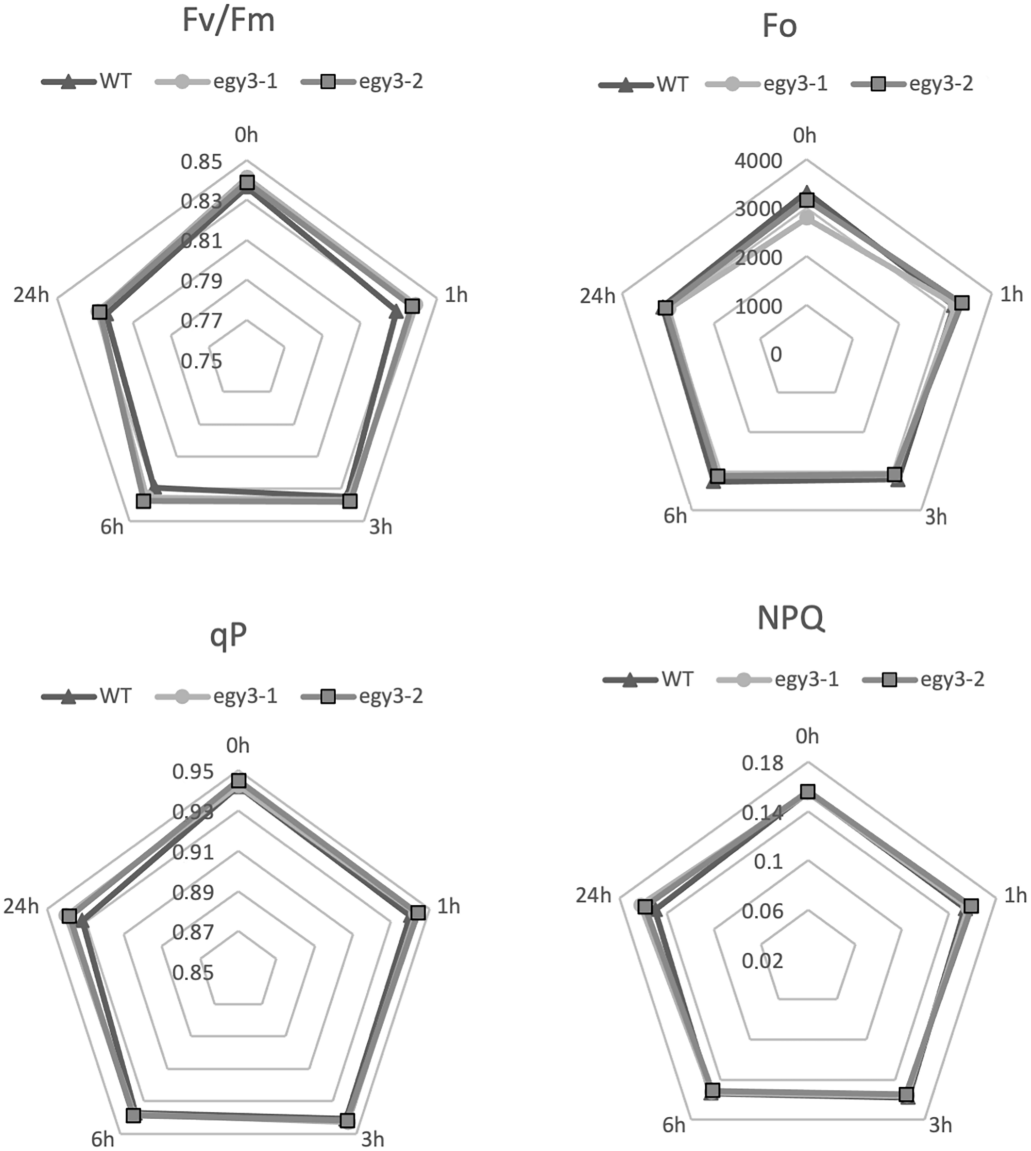
Comparison of chlorophyll fluorescence parameters in wild-type (WT) plants and *egy3* mutants during exposition to high-temperature stress. The presented values are average from three biological replicates (10 plants each).

**Figure 11:**
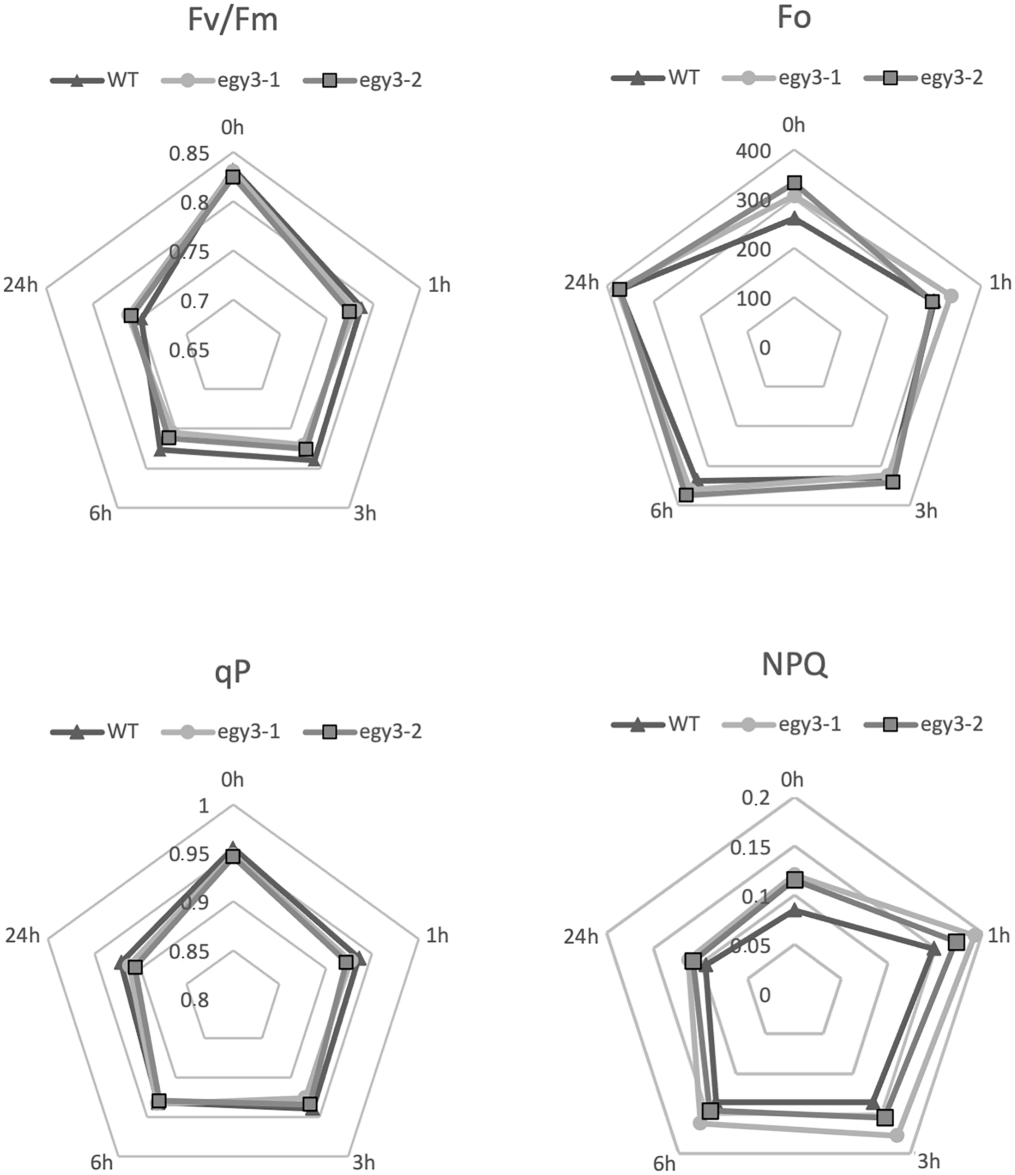
Comparison of chlorophyll fluorescence parameters in wild-type (WT) plants and *egy3* mutants during exposition to high light stress. The presented values are average from three biological replicates (10 plants each).

It is well known that both high light and high-temperature stresses cause severe damage to PSII (Yamamoto, 2016). The dramatic changes in EGY3 transcript and protein abundance during both stresses prompt us to investigate the functional status of PSII in *egy3* mutants. The measurements performed with the PAM fluorescence technique included minimum fluorescence yield (F_o_), the maximum quantum yield of PSII (F_v_/F_m_), photochemical quenching (qP), and non-photochemical quenching (NPQ). Unexpectedly, the changes observed in the analyzed parameters were hardly noticeable. The high-temperature stress did not result in any significant differences in values of analyzed parameters between *egy3* mutant lines and WT plants (Figure 10). During the exposition to high light, in *egy3* mutant lines, we did not observe any significant differences in qP parameter values concerning WT plants (Figure 11). The values of minimum fluorescence yield and maximum quantum yield of PSII in *egy3* mutants were similar to those observed in WT plants in most analyzed time variants. The most differentiating parameter was the NPQ since significant differences between both *egy3* mutant lines and wild-type plants were observed in three of the analyzed time points – 0, 1, and 3 h (Figure 10; Table 3).

**Table 3:** Statistically significant changes of NPQ parameters during high light stress.

Time-variants	WT	*egy3-1*	*egy3-2*
NPQ			
0 h	0.085 ± 0.010	0.121 ± 0.040*	0.116 ± 0.035*
1 h	0.149 ± 0.041	0.192 ± 0.072*	0.172 ± 0.027*
3 h	0.136 ± 0.030	0.177 ± 0.062*	0.155 ± 0.041*

The asterisks indicate statistically significant differences between the WT and individual mutant lines.

## Discussion

### The accumulation of *EGY3* transcript and protein during high-temperature and high light stresses

The performed experiments indicated that the *EGY3* transcript drastically over accumulates after 1 and 3 h of exposition to high-temperature stress. This observation is consistent with previous results, where 16 days old *A. thaliana* plants were exposed to a high temperature which resulted in a significant increase in the abundance of *EGY3* transcript both in roots and shoots (Kilian et al., 2007). In that experiment, however, the temperature stress was applied only for 3 h and then plants were transferred to 25°C for recovery. Our results showed that the longer, 24 h, exposition to high-temperature stress is also accompanied by increased *EGY3* transcript abundance. These results indicate that transcription of *EGY3* increases not only in response to short-term high-temperature stress but is also maintained, however, to a lesser degree during long-term exposition to elevated temperature. The transcript levels, however, do not always correlate well with changes in protein abundance. The relationship between the protein accumulation and the level of a transcript was proven to depend on *inter alia* localization and the function of the protein (Baerenfaller et al., 2012; Liu et al., 2016). Our results provide data confirming the abundance of EGY3 protein increases most significantly after 1 h of exposition to high-temperature stress and it remains elevated also after 3 and 6 h of exposition to high temperature, which correlates well with changes observed at the transcriptional level. The 24-h exposition to 40°C leads, however, to a decrease in EGY3 protein level concerning the one observed in control conditions. This is inconsistent with changes in transcript abundance which remains elevated after 24 h of high-temperature stress. Based on these data, it can be assumed that the physiological role of EGY3 is associated with short-term exposure to high-temperature stress.

The changes in the transcription level of *EGY3* in response to high light stress were not investigated so far. Our results indicate that also during this stress after 1 and 3 h of exposition, the increased abundance of *EGY3* transcript is observed. This overaccumulation correlates with protein abundance which was significantly increased after 1, 3 and 6 h of exposition to high irradiance. Thus, EGY3 protein seems also to play an important role in response to short-term exposure to high light stress.

It was shown that the EGY3 pseudoprotease participates in the stabilization of a chloroplastic copper/zinc superoxide dismutase (CSD2) in response to salt stress (Zhuang et al., 2021). Our results indicate that in response to high light stress, the presence of EGY3 pseudoprotease reassures a higher abundance of CSD2. This result correlates with hydrogen peroxide concentrations, which are lower in *egy3* mutant exposed to analyzed stress conditions. These findings agree with the previous findings concerning the EGY3 role in the salt-stress response (Zhuang et al., 2021). The exposition to high-temperature stress, however, did not cause any statistically significant differences in the abundance of CSD2 protein in *egy3* mutants; however, the lower, than in WT plants, the concentration of hydrogen peroxide was observed. This inconsistency may suggest that in the case of temperature stress, there is a different, CDS2-independent mechanism, leading to lower H_2_O_2_ concentrations and/or a lower activity of CSD2 itself.

### The proteins accumulated differently in response to high light stress

Among proteins identified as those whose accumulation level, in response to high light stress, may be dependent on EGY3 were proteins involved in light-dependent photosynthetic reactions. Three of these proteins: ATPE, PDE334, and NDH-M were identified as overaccumulating. The ATPE and PDE334 proteins were predicted to be involved in ATP synthesis (Berardini et al., 2015), while NDH-M is a subunit of the NDH complex involved in cyclic electron transport within PSI (Rumeau et al., 2005) and alleviating of oxidative stress (Peng et al., 2011). In turn, the abundance of PsaB which is the core protein of PSI was decreased. The proteins CPN60A and CPHSC70-2 (*At5g49910*), which were also identified as overaccumulating in *egy3 A. thaliana* mutant lines, were described as participating in protein folding (Berardini et al., 2015). The CPN60A and CPHSC70-2 (*At5g49910*) have, according to the STRING database (Szklarczyk et al., 2018), relatively high coexpression scores (0.588) not only in *A. thaliana* but also in other organisms (0.572). What is more, both proteins were proven to interact with GUN1, a pentatricopeptide-repeat protein that plays a crucial role in the regulation of plastid development and chloroplast-to-nucleus retrograde communication (Colombo et al., 2016). Moreover, CPN60A was proven to participate in RubisCO folding (Gutteridge and Gatenby, 1995). Another overaccumulating protein was glycine decarboxylase P-protein 1 GLDP1. The protein is a subunit of glycine decarboxylase (GDC), which in mitochondria plays a central role in photorespiration (Bauwe et al., 2010), but its presence in chloroplast was also experimentally confirmed (Zybailov et al., 2008; Ferro et al., 2010). However, the function of GLDP1 in chloroplasts remains unknown.

### The proteins accumulated differently in response to high-temperature stress

In the protein spots with an abundance increase in response to high temperature, two proteins involved in photosynthesis were identified, namely: PSAD-2, ATPF. The PSAD-2 is associated with PSI protein characterized by very high homology to PSAD-1, which is the core protein of PSI. The ATPF, in turn, is one of the subunits of the chloroplast ATPase complex. Another protein overaccumulating in response to high temperature was ferrochelatase II (ATFC-II), which is thought to be involved in the retrograde regulation of photosynthesis-associated nuclear genes (Woodson et al., 2011). In the spot with a lower accumulation level, the RubisCO accumulation factor was identified. The protein was proven to participate in proper RubisCO assembly (Gruber and Feiz, 2018). Also, triosephosphate isomerase (TPI), one of the enzymes crucial for sugar metabolism, was found to be less abundant in *egy3* mutants, as well as glycine decarboxylase P-protein 1 (GLDP1) protein. In turn, glycine decarboxylase P-protein 2 (GLDP2) was identified in the spot which was absent in WT electropherograms but repeatedly appeared in *egy3* mutants. Similar to GLDP1, GLDP2 is a subunit of the mitochondrial GDC complex, but its presence in chloroplast was previously confirmed experimentally (Ferro et al., 2010). In another spot absent in WT present in *egy3* electropherograms, ACR11 protein was identified. The expression of ACR11 and GLN2 was found to be highly correlated, and the ACR11 was suggested to participate in glutamine metabolism or sensing in *Arabidopsis* (Sung et al., 2011).

## Conclusion

The significant accumulation of the *EGY3* transcript as well as the protein itself indicates that EGY3 participates in response to both high-temperature and high light stresses. Since the protein participates in the regulation of hydrogen peroxide content, probably *via* stabilization of CSD2 protein, it can be considered as part of a retrograde chloroplast-nucleus signaling pathway. It remains unclear, however, which chloroplast pathways and processes are regulated in an EGY3-dependent manner. The analysis of the functional status of PSII during high light and high-temperature stresses indicates, however, no significant differences between *egy3* mutants and WT plants. Also, no proteins related to PSII were identified as accumulated differently in *egy3* mutants. This indicates that EGY3 protein does not play a significant function in maintaining the proper functioning of the PSII. The proteins identified as accumulated differently in both analyzed stresses participate in the same processes as RubisCO folding or glycine metabolism. In both stresses, proteins related to PSI were also identified as accumulated differently in *egy3* mutants. This is consistent with previous suggestions that EGY3 may interact with PSI subunits (Zhuang et al., 2021). The results suggest that future experiments aimed to determine the physiological role of EGY3 should be focused rather on PSI and light-independent reactions.

## Data availability statement

The original contributions presented in the study are included in the article, further inquiries can be directed to the corresponding author.

## Author contributions

MA: developed the article concept, participated in the 2D experiments, fluorescence measurement, data analysis, and drafted the article. JD: performed the RT-PCR experiments and cooperated in hydrogen peroxide detection experiments. ŁW: cooperated in hydrogen peroxide detection experiments. RL: cooperated in developing the concept of paper, participated in the design and realization of experiments, and helped in the data analysis. All authors contributed to the article and approved the submitted version.

## Funding

This work was supported by the National Science Center, Poland based on decision number: dec-2019/03/X/NZ3/00303.

## Conflict of interest

The authors declare that the research was conducted in the absence of any commercial or financial relationships that could be construed as a potential conflict of interest.

## Publisher’s note

All claims expressed in this article are solely those of the authors and do not necessarily represent those of their affiliated organizations, or those of the publisher, the editors and the reviewers. Any product that may be evaluated in this article, or claim that may be made by its manufacturer, is not guaranteed or endorsed by the publisher.
